# Drinking water pollution with respective of fluoride in the semi-arid region of Basara, Nirmal district, Telangana State, India

**DOI:** 10.1016/j.dib.2017.11.087

**Published:** 2017-12-06

**Authors:** Adimalla Narsimha, Venkatayogi Sudarshan

**Affiliations:** Department of Applied Geochemistry, University College of Science, Osmania University, Hyderabad 500007, India

## Abstract

Fluoride is an essential microelement for human health. Statistically, smaller quantities (<1.0 mg/L) in drinking water are usually considered to have a beneficial effect on the rate of occurrence of dental caries, particularly among children, but excessive continuous exposure (>1.5 mg/L) to fluoride can give rise to a number of adverse effects, including dental fluorosis, skeletal fluorosis, increased rate of bone fractures, decreased birth rates, increased rate of urolithiasis (kidney stones), impaired thyroid function, and impaired development of intelligence in children [1], [2], [3], [4], [5]. The data suggested that the north-eastern part of the Basara region having high fluoride concentration, which is unsuitable for drinking purposes. Hence, this unsuitable drinking water cause fluorosis in this Basara and surrounding villages, and especially based on the findings suggests, where the fluoride levels are in below maximum permissible limits that water ingests to the people to avoid further fluorosis.

**Specifications Table**

**Table:** 

Subject area	*Earth Science*
More specific subject area	*Hydro-geochemistry*
Type of data	*Table and figure*
How data was acquired	*Thermo Scientific Orion Star A214 Benchtop pH/ISE meter*
Data format	*Analyzed*
Experimental factors	*Groundwater samples were collected after pumping the wells for 5–10 min and rinsing the 1.0 l polyethylene bottles for two to three times with water to be sampled.*
Experimental features	*Determine the content levels of fluoride*
*Data source location*	*Location: Basara, Region: Nirmal, State: Telangana, India*
	*Topo-sheet: 56F/13, GPS: North latitudes 18° 51' and 18° 59' and East longitudes 77° 54' and 77° 59'*
	*Covering an areas: 186 Sq. Km.*
Data accessibility	*Data is with this article.*

**Value of the Data**

• *Estimation of fluoride concentration in waters is benchmark for identification of vulnerability and safe zones.*
• *Data on fluoride levels provide an idea to continue a further research in this aspect and has importance in especially in rural areas, where there is no proper drinking facility.*
• *In spite of having water in abundance, it is of no use due to presence of excess of fluoride concentration. Identification of safer zones will mitigate the fluoride problem.*
• *It could be used in future for the better understanding of groundwater quality assessment and will provide baseline data for further studies.*

## 1 Data

It is understood that the fluoride concentration levels are ranged from 0.06 to 4.33 mg/L, with a mean of 1.13 mg/L (Table 1). Fluoride data are shown in the form of distribution map (Fig. 2) and it reveals north-eastern part of the region having higher concentration of fluoride, which is not fit for drinking purposes. Mutual relationship of fluoride and other chemical elements are depicts in Fig. 3.

**Fig. 1:**
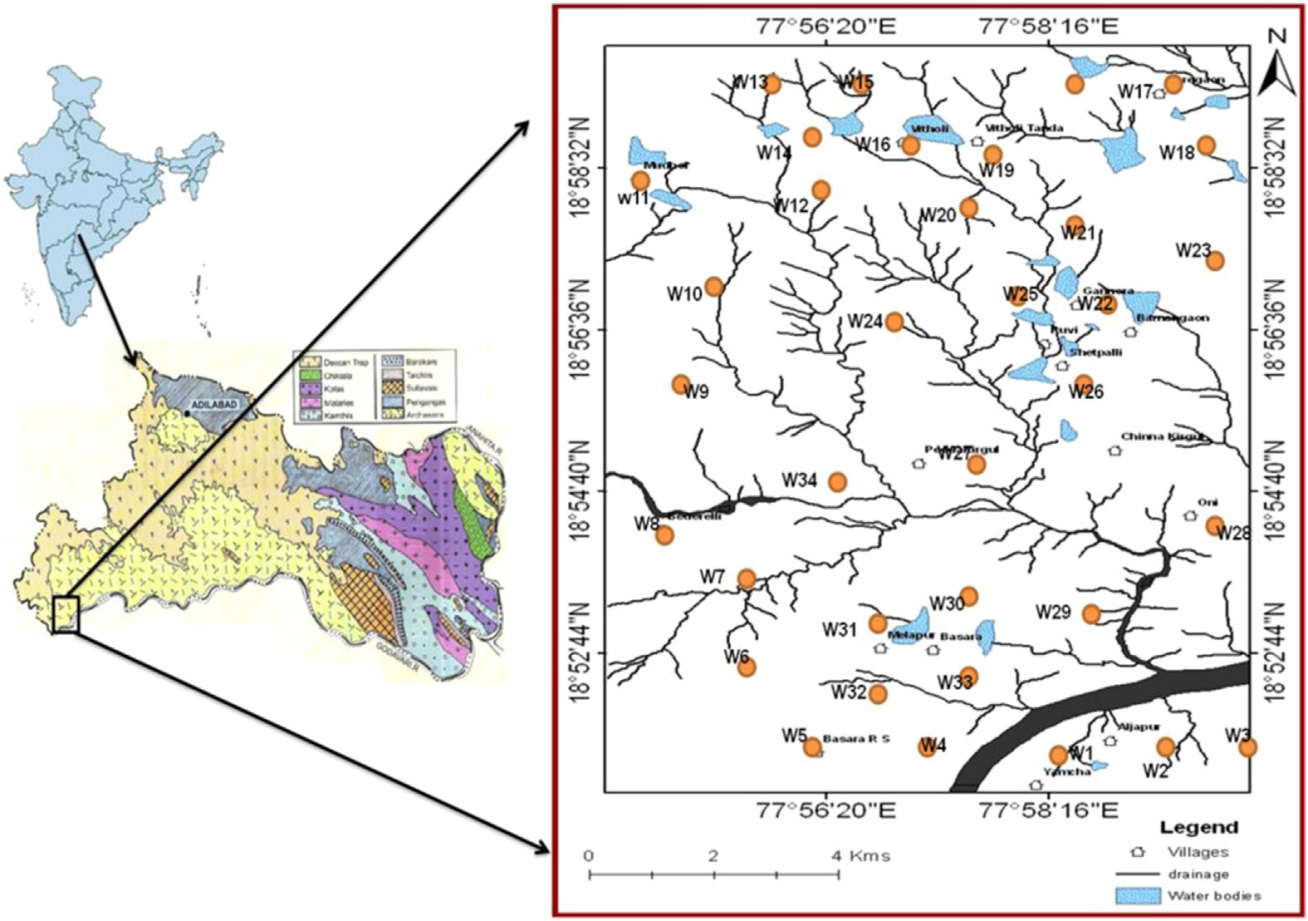
Location map of the Basara area and its groundwater locations along with drainage pattern from Nirmal district, Telangana State, South India.

**Fig. 2:**
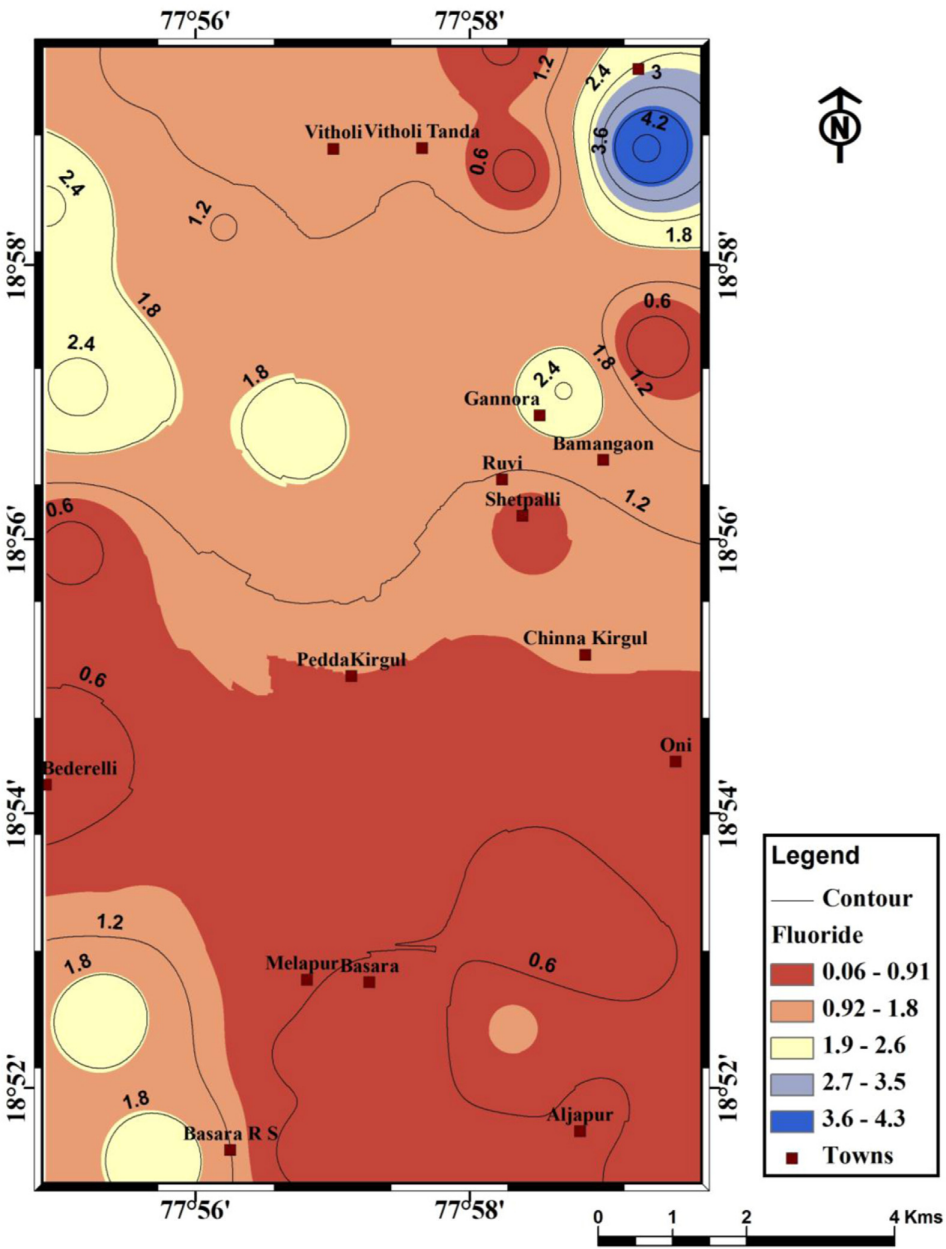
Spatial distribution of fluoride in Basara region, Nirmal district, Telangana State, *South India*.

**Fig. 3:**
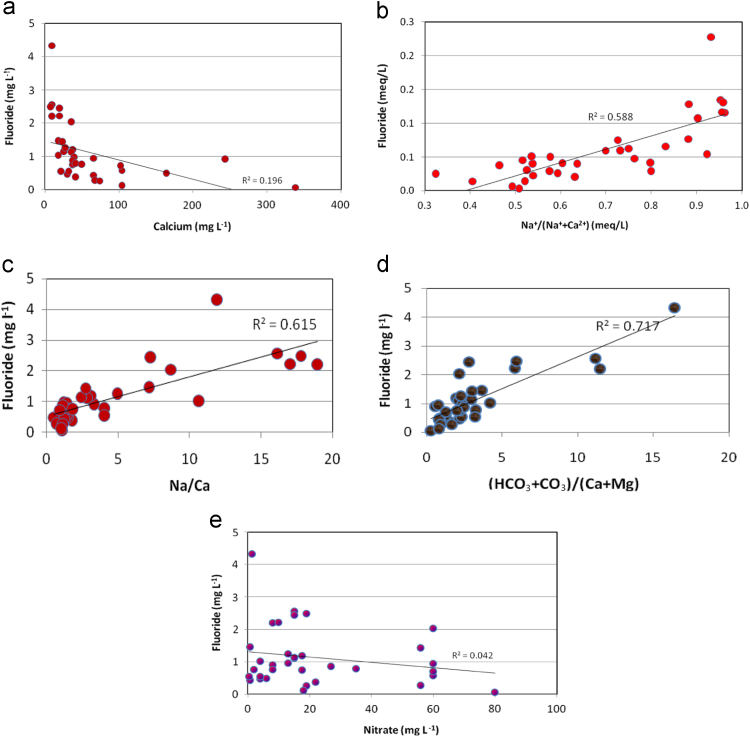
Mutual relationship of fluoride and Calcium, Na^+^/(Na^+^ + Ca^2+^), ($HC{O_{3}}^{-}+C{O_{3}}^{2-}$ )/(Ca^2+^ + Mg^2+^), Nitrate.

**Table 1:** Statistical parameters, BIS, 1991 limits and the percentage of groundwater samples exceeding the prescribed limits for drinking water.

Constituents (mg/L)	Min.	Max.	Mean	Std. Dev	Permissible Limit	% of samples exceeding the limit
pH	6.47	8.03	7.09	0.4	8.5	0
TDS	150	1355	377.82	275.51	2,000	0
TH	35	850	197.35	172.61	600	5.88
Ca^2+^	8	339	58.97	68.8	200	5.88
Mg^2+^	1	29	11.94	7.28	100	0
Cl-	42	1010	182.06	197.43	1,000	3
$SO_{4}^{2-}$	2.5	375	56.99	85.48	600	0
$N{O_{3}}^{-}$	0.4	80	22.07	22.18	45	20
F^−^	0.06	4.33	1.13	0.9	1.5	20

## 2 Experimental design, materials, and methods

### 2.1 The study area description

The present study area is located in south-western part of the Nirmal district and forms part of the Survey of India toposheet 56F/13. The area lies between the North latitudes 18° 51' and 18° 59' and East longitudes 77° 54' and 77° 59' covering an areas of 186 Sq. Km (Fig. 1).

### 2.2 Sample collection and analytical procedures

34 groundwater samples were collected systematically during the month of May, 2011 from bore/hand pumps of the study area. The digital pH systronic 802 m was first calibrated with buffer solutions of pH 4.0, 7.0, and 9.0 and then pH of samples were determined. The electrical conductivity (Systronics, 304) meter was calibrated with standard KCl solution (0.1 M). Total dissolved solids (TDS) were computed as per [6], [7] from EC values multiplied by 0.64. Calcium (Ca^2+^) and Magnesium (Mg^2+^) were determined titrimetrically using standard EDTA. Sodium (Na^+^) and Potassium (K^+^) concentrations were determined using Flame photometer (Systronics, 130). Sulphate ($SO_{4}^{2-}$ ) and Nitrate ($N{O_{3}}^{-}$) were determined by using UV-visible spectrophotometer (Spectronic, 21, BAUSCH and LOMB). Fluoride (F^−^) was estimated by using an ion-selective electrode (ISE) with Orion 4 star meter benchtop pH/ISE meter.
